# Prevalence and pattern of dental anomalies among schoolchildren in Aden, Yemen

**DOI:** 10.1186/s12903-026-09644-1

**Published:** 2026-08-18

**Authors:** Omar Abdullah Rageh, Ali N. M. Gubran, Abdullah N. Yousef, Nasser Omar Jama, Ghazah. A. Ahmed, Aamer A. Gabr, Ali M. Ali, Ahmed B. Abdo Alaredha, Toga N. Algabery, Fadhl R. AL-Sanbi

**Affiliations:** 1 Department of Dentistry, Faculty of Medicine and Health Sciences, University of Science and Technology, Aden, Yemen; 2 Department of Health Sciences, Faculty of Medicine and Health Sciences, University of Science and Technology, Aden, Yemen

**Keywords:** Dental anomalies, Tongue tie, Developmental anomalies, Pediatric dentistry, BMI, Yemen

## Abstract

**Background:**

Dental anomalies (DAs) are developmental variations affecting oral health and quality of life. Epidemiological data on DAs in pediatric populations affected by conflict remain scarce.

**Objectives:**

To determine the prevalence and pattern of DAs and evaluate associated demographic and nutritional factors among schoolchildren in Aden, Yemen.

**Methods:**

A cross-sectional study was conducted among 1,026 schoolchildren aged 8–13 years in Aden. Clinical examinations identified DAs, and nutritional status was classified using WHO 2007 BMI z-scores. Statistical analysis utilized chi-square tests and cluster-adjusted multivariate logistic regression with Bonferroni correction (alpha = 0.007).

**Results:**

The overall DA prevalence was 29.6% (*n* = 304; 95% CI: 26.8–32.4%). DAs were significantly more common in children aged < 10 years (34.1%), males (40.9%), and those with low BMI (69.4%) (*p* < 0.001 for all). Tongue tie was the most frequent anomaly (11.6%), occurring predominantly in males (68.1%, *p* = 0.001) and younger children (52.1%, *p* = 0.005). In multivariate analysis, age > 11 years {AOR} = 0.404; 95% CI: 0.276–0.592; *p* < 0.001) and female sex {AOR} = 0.319; 95% CI: 0.238–0.427; *p* < 0.001) were protective factors, whereas low BMI was a strong risk factor {AOR} = 4.13; 95% CI: 1.8–9.5; *p* = 0.001).

**Conclusion:**

The prevalence of DAs, especially tongue tie, was high among school children in Aden. DAs were strongly linked to younger age, male gender and low BMI. These results highlight a substantial requirement for oral health services in this population, but the absence of radiographic confirmation restricts lower-bound estimates for some anomalies.

## Introduction

Dental anomalies (DAs) are clinical deviations from normal odontogenesis, encompassing variations in tooth number, size, shape, position, and structure [1]. These conditions extend beyond aesthetic concerns, frequently contributing to malocclusion, increased caries susceptibility, and periodontal disease, thereby significantly impacting the oral health-related quality of life (OHRQoL) in pediatric populations [2]. While the prevalence of DAs varies globally, ranging from 10% to over 30% due to complex gene-environment interactions [3], recent meta-analyses indicate a pooled prevalence of 2.6% to 45.1% in Middle Eastern populations [4].

Yemen’s epidemiological profile, however, is a unique and critical one. A long and deep humanitarian crisis, together with the unique DNA of the nation, has created an environment of extreme systemic stress. Given the high rates of malnutrition (> 45%) [5], the international dental standards may not be suitable for the clinical situation of Yemeni children [6]. This context is further emphasized by a recent study from Sana’a University, which reported a prevalence of dental anomalies of 32.3% in a Yemeni pediatric cohort, with shape anomalies (61.8%) and tooth agenesis (17.5%) being particularly frequent [7]. The high frequency of anomalies such as taurodontism suggests a possible influence of genetic factors specific to the region [7].

Despite this alarming context, a significant knowledge gap persists regarding the association between nutritional status, as measured by the Body Mass Index (BMI), and the occurrence of dental anomalies. While environmental stressors, such as undernutrition, are known to cause enamel defects and eruption delays [8], the theory of genetic canalization posits that essential developmental processes, including aspects of tooth formation, may be robust against such external perturbations [9]. This hypothesis suggests that certain anomalies, like hypodontia, may be more genetically buffered and thus less susceptible to nutritional stress than others [9]. Furthermore, healthcare systems in conflict-affected, resource-limited settings like Yemen face the critical challenge of prioritizing clinical cases for specialist referral. Currently, validated, data-driven models to stratify dental anomalies by clinical urgency are lacking, hampering efficient resource allocation and optimal patient care [6].

The term “Yemen Paradox” is tentatively proposed to describe a potential phenomenon in which high nutritional stress is associated with a differential pattern of dental anomalies, with some anomalies being affected while others remain relatively spared. This may be due to the differential susceptibility of different aspects of odontogenesis to environmental stressors, with the development of tooth number (e.g., hypodontia) being relatively canalized compared to size and positional anomalies [9]. We emphasize that this is a preliminary hypothesis that requires confirmation through radiographic and genetic studies. The present study is an attempt to fill these gaps by offering a comprehensive analytical map of dental anomalies in schoolchildren in Aden, Yemen. The objectives are (1) to determine the prevalence of dental anomalies in this population and (2) to identify the contributions of age, sex, and BMI to the occurrence of dental anomalies.

## Materials and methods

Yemeni primary school students in the Aden Governorate participated in this cross-sectional epidemiological study. To capture the varied socioeconomic landscape of the post-conflict urban environment, data were collected between January and May 2025 at different representative schools. Children aged 8–13 years who were enrolled in participating schools and had signed consent forms from their parents were eligible for inclusion. Children with a history of extensive orthodontic treatment, those with systemic syndromes (such as Down syndrome or ectodermal dysplasia), or those who were uncooperative during the clinical examination were excluded.

Hypodontia and impactions were evaluated by clinical examination only, i.e., by the absence of teeth beyond the normal eruption window (± 2 years), as radiographs were not taken in the field due to logistical limitations in the conflict-affected setting. However, it is worth noting that, in the absence of radiographic confirmation, conditions such as hypodontia, supernumerary teeth, and impactions are likely to be under-reported or misclassified, as these anomalies are usually diagnosed by radiography in clinical practice.

### Sample size

Based on a previous study and using Stephen Thompson’s formula, the minimum required sample size was 528, calculated using a 95% confidence level, a 5% margin of error, and an estimated DA prevalence of 20.1% from previous regional studies [10]. The final sample was increased to 1,026 schoolchildren after adjusting for a non-response rate (20%) and a design effect associated with multistage cluster sampling technique was used to account for district-level variation and to boost the power of multivariate analysis.

### Clinical examination and diagnostic criteria

Prior to field examinations, the two pediatric dentists underwent a standardization and calibration session using clinical photographic reference sets. Diagnostic consistency was informally evaluated during a pilot trial of 30 non-sampled schoolchildren, achieving over 90% diagnostic agreement prior to formal data collection. Impacted teeth: Failure of tooth eruption beyond the normal eruption window (± 2 years), with clinical evidence of the tooth in the arch or adjacent swelling; depth of impaction was not assessed due to lack of radiographs. Diagnostic criteria were aligned with updated expert consensus guidelines [4]. Talon cusp was defined strictly as an accessory cusp projection on the cingulum/CEJ of anterior teeth, explicitly distinguishing it from Carabelli traits on molars. Peg-shaped laterals were classified based on classical conical morphology, where incisal width was reduced relative to normal cervical width. Macrodontia was evaluated as clinical macrodontia, defined as a mesiodistal crown diameter SD above population norms relative to adjacent teeth, acknowledging that root dimensions could not be radiographically verified [11].

Nutritional status was assessed using BMI z-scores based on WHO 2007 growth references for children aged 5–19 years. Children were classified as having low BMI (z-score < -2 SD), normal BMI (-2 SD to + 1 SD), or high BMI ( > + 1 SD) [12].

### Statistical analysis

Data were entered and analyzed using the Statistical Package for the Social Sciences (SPSS^®^) software (Version 26). Children’s ages were normally distributed; therefore, mean and standard deviation (SD) values were determined. Qualitative data were presented as frequencies and percentages. Significant associations between different variables were determined by calculating p-values using the chi-square (χ²) test, where a p-value ≤ 0.05 indicated statistical significance. Multivariable logistic regression was used to assess the impact of independent variables and to control for confounders. To account for the multistage cluster sampling design, we first calculated the intra-cluster correlation coefficient (ICC) for the overall prevalence of dental anomalies at the school level. The ICC was exceptionally low (ICC < 0.015), indicating negligible clustering effects and a design effect close to unity. Nevertheless, to ensure statistical rigor and fully adjust for the sampling design, we re-fitted the multivariable logistic regression model using Generalized Estimating Equations (GEE) with an exchangeable correlation structure and cluster-robust standard errors, clustered at the school level. This approach accounts for potential dependence of observations within the same school and provides valid inference. All reported Adjusted Odds Ratios (AORs), 95% Confidence Intervals, and p-values in Table 6 are derived from this cluster-adjusted model.

## Results

A total of 1,026 schoolchildren who met the inclusion criteria were included in the study, consisting of equal numbers of male and female children (513 each, 50%). The mean ± SD age was 10.54 ± 1.44 years, with most participants (399, 38.9%) falling below the age of 10 years. The majority (755, 73.6%) had low BMI (Table 1).

**Table 1:** General characteristics of schoolchildren in Aden, Yemen (*N* = 1026)

Category	Frequency	Percentage (%)
Age group years		
< 10	399	38.9
10–11	323	31.5
> 11	304	29.6
(Mean ± SD)	10.54 ± 1.44	
Sex		
Male	513	50.0
Female	513	50.0
BMI		
Normal	213	20.8
Low	755	73.6
High	58	5.7

**Fig. 1:**
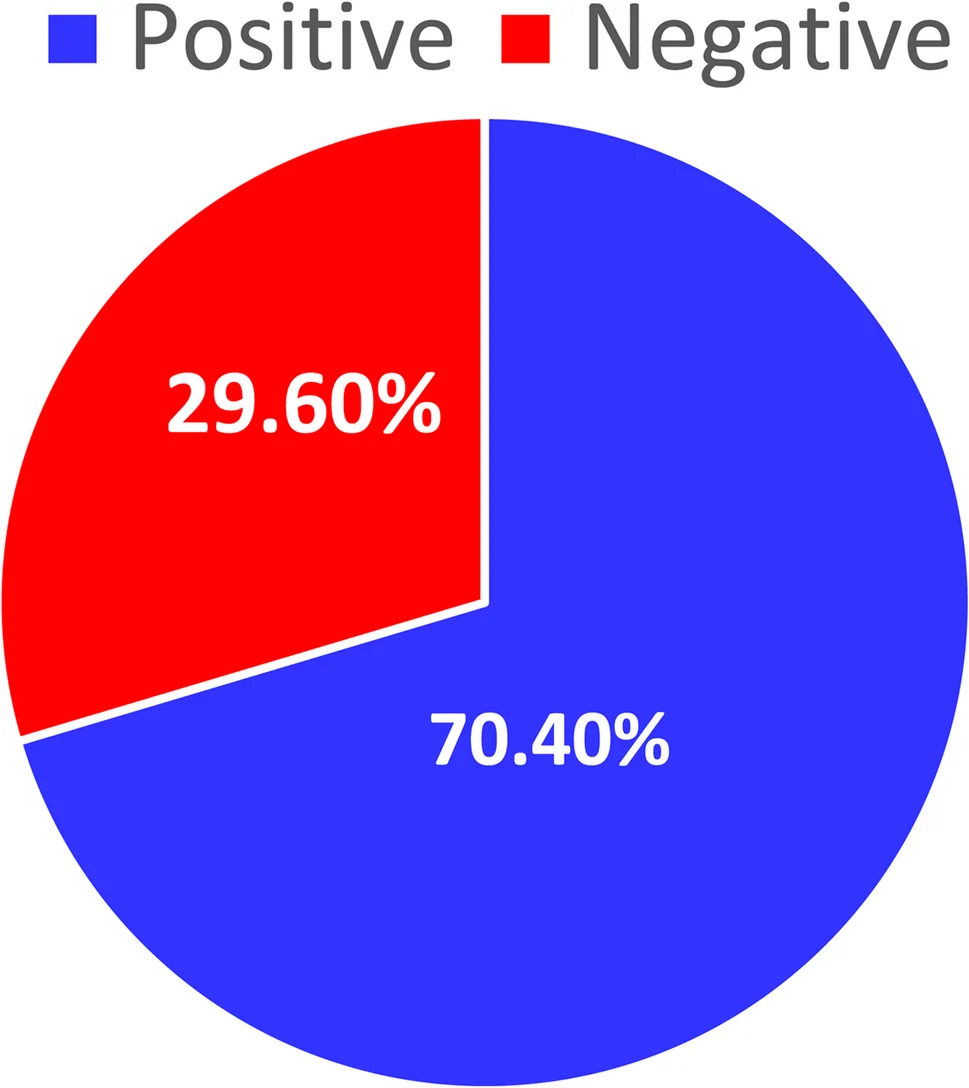
Prevalence of dental anomalies among schoolchildren in Aden, Yemen (*N* = 1026)

The overall prevalence of dental anomalies among children was 304 (29.6%) (Fig. 1). The highest rates were 136 (34.1%) in the age group less than 10 years, 210 (40.9%) among male children, and 211 (69.4%) among those children who had low BMI. All these results were highly statistically significant (*P* < 0.001), each (Table 2).

Although bivariate associations were initially examined using chi-square tests without cluster adjustment, the exceptionally low intra-cluster correlation coefficient (ICC < 0.015) confirmed that school-level clustering had a negligible effect on the estimates. Therefore, the unadjusted chi-square tests provide a valid approximation of the bivariate associations. All multivariate findings and conclusions are based on the cluster-robust Generalized Estimating Equations (GEE) model, which fully accounts for the multistage sampling design.

**Table 2:** Prevalence of dental anomalies among schoolchildren by their demographic characteristics (*N* = 1026)

Category	Present of dental anomalies	Prevalence (%)	*P* - value
Age group years			
< 10 (*n* = 399)	136	34.1	< 0.001
10–11 (*n* = 323)	104	32.2	
> 11 (*n* = 304)	64	21.1	
Sex			
Male (*n* = 513)	210	40.9	< 0.001
Female (*n* = 513)	94	18.3	
BMI			
Normal (*n* = 213)	85	28.0	< 0.001
Low (*n* = 755)	211	69.4	
High (*n* = 58)	8	2.6	

*P* < 0.05 is considered significant

**Fig. 2:**
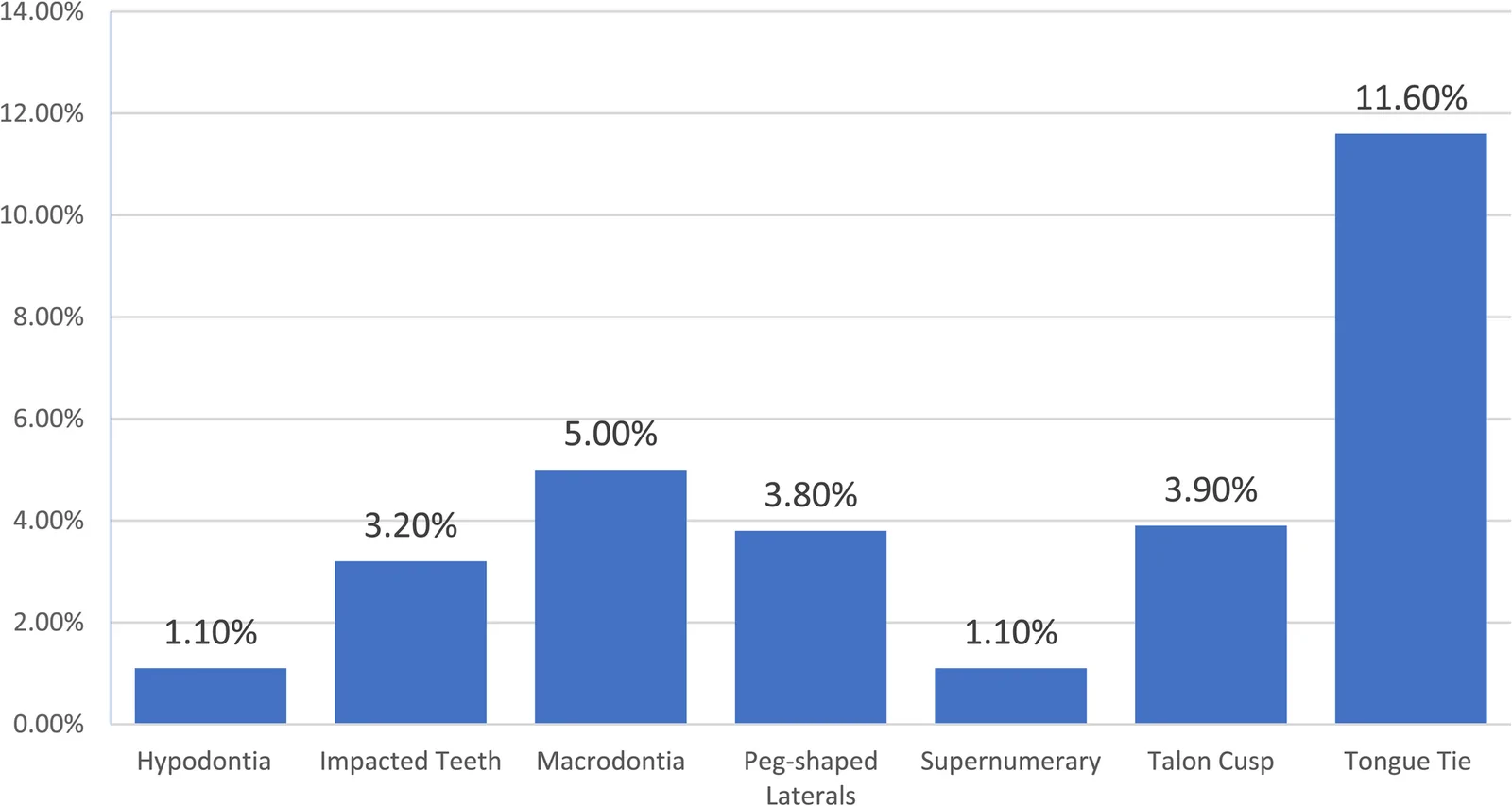
The prevalence of specific dental anomalies among schoolchildren in Aden, Yemen (*N* = 1026)

The most frequent anomaly among schoolchildren was tongue tie, found in 119 (11.6%) children, followed by macrodontia in 51 (5.0%) children (Fig. 2). Tongue tie occurred significantly more often in males (68.1%, *P* = 0.001) (Table 3).

**Table 3:** The prevalence specific dental anomalies among schoolchildren by their sex in Aden, Yemen (*N* = 1026)

Dental Anomaly	Male		Female		*P* - value
	*n*	%	*n*	%	
Hypodontia (*n* = 11)	3	27.3	8	72.7	0.112
Impacted Teeth (*n* = 33)	12	36.4	21	63.6	0.078
Macrodontia (*n* = 51)	21	41.2	30	58.8	0.125
Peg-Shaped Laterals (*n* = 39)	19	48.7	20	51.3	0.500
Supernumerary (*n* = 11)	5	45.5	6	54.5	0.500
Talon Cusp (*n* = 40)	16	40.0	24	60.0	0.129
Tongue Tie (*n* = 119)	81	68.1	38	31.9	0.001

*n *number of cases

*P* < 0.05 is considered significant

According to age, the results indicated a significantly higher prevalence of impacted teeth, supernumerary teeth, and tongue tie in the youngest age group (children aged below 10 years): 90.9%, 72.7%, and 52.1%, respectively (*p* < 0.001, 0.01, and 0.005, respectively). Conversely, hypodontia, macrodontia, and talon cusp were significantly associated with children in the age group 10–11 years: 90.9%, 62.7%, and 57.5%, respectively (*p* < 0.001, < 0.001, and 0.001, respectively) (Table 4).

**Table 4:** The Prevalence of dental anomalies by age group among school children in Aden (*N* = 1026)

Dental Anomaly	Age group years						*P* - value
	Less than 10		10–11		More than 11		
	*n*	%	*n*	%	*n*	%	
Hypodontia (*n* = 11)	0	0.0	10	90.9	1	9.1	< 0.001*
Impacted Teeth (*n* = 33)	30	90.9	2	6.1	1	3.0	< 0.001*
Macrodontia (*n* = 51)	14	27.5	32	62.7	5	9.8	< 0.001
Peg-Shaped Laterals (*n* = 39)	20	51.3	10	25.6	9	23.1	0.269
Supernumerary (*n* = 11)	8	72.7	3	27.3	0	0.0	0.01*
Talon Cusp (*n* = 40)	12	30.0	23	57.5	5	12.5	0.001
Tongue Tie (*n* = 119)	62	52.1	26	21.8	31	26.1	0.005

*n* number of cases

*P* < 0.05 is considered significant

*Fisher’s exact test

Regarding the BMI of children, this data noticed that all eleven children (100%) who has supernumerary teeth had low BMI (*P* = 0.034). The talon cusp and tongue tie were significantly associated with low BMI (*P* = 0.024 and 0.007), respectively (Table 5).

**Table 5:** The prevalence specific dental anomalies among schoolchildren by their BMI in Aden, Yemen (*N* = 1026)

Dental Anomaly	BMI						*P* - value
	Normal		Low		High		
	*n*	%	*n*	%	*n*	%	
Hypodontia (*N* = 11)	2	18.2	9	81.8	0	0.0	0.500*
Impacted Teeth (*N* = 33)	3	9.1	29	87.9	1	3.0	0.122*
Macrodontia (*N* = 51)	10	19.6	39	76.5	2	3.9	0.827*
Peg-Shaped Laterals (*N* = 39)	10	25.6	26	66.7	3	7.7	0.614*
Supernumerary (*N* = 11)	0	0.0	11	100.0	0	0.0	0.034*
Talon Cusp (*N* = 40)	3	7.5	32	80.0	5	12.5	0.024*
Tongue Tie (*N* = 119)	17	14.3	100	84.0	2	1.7	0.007*

*n* number of cases

*P* < 0.05 is considered significant

*Fisher’s exact test

Multivariate logistic regression analysis revealed that children aged > 11 years had significantly lower odds of dental anomalies compared to those aged < 10 years (AOR = 0.404; 95% CI: 0.276–0.592; p < 0.001). Female sex was a significant protective factor (AOR = 0.319; 95% CI: 0.238–0.427; p < 0.001). Low BMI was associated with a four-fold increased risk of dental anomalies (AOR = 4.13; 95% CI: 1.8–9.5; p = 0.001). After Bonferroni correction for multiple comparisons (α = 0.007), all three associations remained statistically significant (Table 6). Children aged 10–11 years showed no significant difference compared to the reference group (AOR = 0.795; 95% CI: 0.565–1.118; p = 0.188), and high BMI was not significantly associated with dental anomalies (AOR = 1.73; 95% CI: 0.77–3.87; p = 0.182).”

**Table 6:** Multivariate logistic regression analysis of factors associated with the presence of dental anomalies among schoolchildren (*N* = 1026)

Variable	AOR	95% CI	*P* - value	Bonferroni-corrected *P*	Interpretation
Age group					
< 10	1.00	Reference	-	-	Reference
10–11	0.795	0.565–1.118	0.188	0.188	Not significant
11	0.404	0.276–0.592	< 0.001	< 0.001*	Confirmed
Sex					
Male	1.00	Reference	-	-	Reference
Female	0.319	0.238–0.427	< 0.001	< 0.001*	Confirmed
BMI					
Normal	1.00	Reference	-	-	Reference
Low	4.13	1.8–9.5	0.001	0.007*	Confirmed
High	1.73	0.77–3.87	0.182	0.182	Not significant

*AOR* Adjusted Odds Ratio derived from Generalized Estimating Equations (GEE) with cluster-robust standard errors to account for school-level clustering, *CI* 95% Confidence Interval

*p < 0.007 (Bonferroni-corrected for 7 comparisons, α = 0.05/7)

## Discussion

This is the first epidemiological study on dental anomalies that aimed to determine the prevalence of dental anomalies and identify associated factors among schoolchildren in Aden, Yemen, reporting an occurrence of 29.6%, which is among the highest recorded in the literature.

This temporal pattern provides a basis to hypothesize that early developmental periods coincided with times of severe environmental and nutritional hardship in Aden, rather than establishing a direct causal link between conflict exposure and odontogenic disruption.

This rate is significantly higher than that found in other population groups, such as 26.6% in Nigerian schoolchildren [13], 25.8% in Indian schoolchildren [14], 25.4% among Saudi children [15], 39.9% in Brazilian schoolchildren [16], 20.1% in Kuwaiti children [17], 16.7% in Albanian population [18], and 5.2% in Australian children [19]. A study conducted among Nigerian schoolchildren revealed one of the lowest rates at 4.2% [20]. This introduced burden defines the “Yemen Paradox”—where high nutritional stress is associated with some, but not all, dental anomalies—a special epidemiological phenomenon demanding further investigation.

For the current study, the highest significantly high prevalence was recorded for the children under the age of 10 years (*p* < 0.001). There were conflicting results in studies conducted in Kuwait [17]. For this particular study, there was a significant strong inverse association between the age of the children and dental anomalies. The 10-11-year-old children showed a non-significantly high risk of developing dental anomalies compared to children below 10 years (AOR = 0.795; *p* = 0.188). The multivariate analysis confirmed our finding that children aged > 11 years had 60% lower odds of anomalies (AOR = 0.404; 95% CI: 0.276–0.592; *p* < 0.001), supporting the hypothesis that younger children born during intensified conflict periods experienced greater developmental stress. Children under the age of 10 in our study were born and grew up during periods of intensified conflict in Yemen, characterized by widespread maternal malnutrition and stress [18, 19]. This temporal association raises the hypothesis that prenatal and early postnatal exposure to conflict-related stressors may have contributed to the higher prevalence of dental anomalies in this age group. In contrast, children aged over 11 had largely completed the critical periods of tooth formation before the conflict escalated to its most severe stages, which may explain the relatively lower frequency of anomalies among them. However, we acknowledge that this interpretation is speculative and cannot be confirmed without longitudinal data on individual nutritional and stress exposures. Future studies with prospective designs and individual-level exposure data are needed to test this hypothesis.

“In terms of sexual distribution, the prevalence rate of DA that was statistically significant was 40.9% in males. Multivariate analysis indicated that being female was significantly associated with protection against dental abnormalities (AOR = 0.319; 95% CI: 0.238–0.427; p < 0.001). In contradiction to previous studies conducted in Kuwait and Nigeria that found higher DA prevalence in females [17, 20], these results indicate that environmental factors have different effects on odontogenesis in females and males in Yemen. Males have been described as more biologically vulnerable during certain developmental periods according to the concept of “male fragility” [21].

Our research revealed that low BMI subjects exhibited the highest DA prevalence rate (69.4%). Low BMI remained a significant risk factor even after conservative Bonferroni correction (AOR = 4.13; 95% CI: 1.8–9.5; *p* = 0.001; corrected *p* = 0.007), reinforcing the association between nutritional status and dental developmental anomalies. This is one of the first empirical studies to provide evidence for this finding.

Tongue tie was the most common DA (11.6%), while macrodontia was the second most common (5.0%). According to research conducted by Olatosi et al., hypoplasia was the most common DA, followed by the retention of a primary tooth [22]. According to Alanzi et al., tooth number anomaly and taurodontism were the most common anomalies, respectively [16]. A Saudi Arabian study found that the most common DA was hypodontia, followed by hyperdontia [23]. The reason for this difference can be related to diagnostic criteria, genetic predisposition, and types and number of anomalies detected.

Concerning a relation between anomalies and sex, ankyloglossia (tongue tie) is the only DA that had a statistical difference between male and female children, with twice more males (68.1%) than females (31.9%, *p* = 0.001). This is highly consistent with findings from Saudi Arabia, where a systematic review demonstrated a male-to-female ratio of approximately 2:1 for ankyloglossia [23]. The physiological explanation for increased risk among males may be associated with their greater vulnerability to prenatal and perinatal stress.

The anomaly-specific analysis (Table 4) indicated a significantly higher prevalence of impacted teeth, supernumerary teeth, and tongue tie among children aged below 10 years (90.9%, 72.7%, and 52.1%, respectively; *p* < 0.001, 0.01, and 0.005, respectively), while hypodontia, macrodontia, and talon cusp were significantly associated with children in the age group 10–11 years (90.9%, 62.7%, and 57.5%, respectively; *p* < 0.001, < 0.001, and 0.001, respectively). These results differ from those reported in Nigeria [22]. These age-specific patterns indicate that the developmental timing of dental anomalies may be affected by population-specific genetic, nutritional, and environmental factors.

The data obtained in our study indicate strong significant association between underweight (low BMI) and certain dental anomalies (Table 5). The prevalence of supernumerary teeth was limited to low-BMI subjects only (100.0%, *n* = 11; *p* = 0.034). The prevalence of talon cusp was also high among low-BMI children (80.0%, *n* = 32; *p* = 0.024). The strongest correlation between low BMI and dental anomaly was identified for tongue tie (84.0%, *n* = 100; *p* = 0.007). Shrestha, et al. (2019) noted that dental developmental anomalies may be associated with nutrition during odontogenesis due to delays in tooth eruption in the low-BMI group [24]. Talon cusp occurs with higher prevalence among children with low growth percentiles, and ankyloglossia causes feeding difficulties, which can lead to nutritional disorders and result in low BMI [25].

The normal prevalence of hypodontia (0.49%) in the presence of extensive malnutrition (73.6% low BMI) indicates that the development of tooth number is genetically determined and resistant to nutritional stress—a phenomenon termed the “Yemen Paradox” for the first time in this setting.

The prevalence of hypodontia in our study (0.49%) is much lower than those reported in radiographic studies worldwide (26–28). This discrepancy likely reflects the lack of radiographic confirmation in our clinical examination rather than a truly lower prevalence in this population. Without imaging we were unable to differentiate true agenesis from delayed eruption, which is particularly concerning as our participants were comprised of children aged 8–13 years, some of whom may not have completed dental development. We note that our estimates of hypodontia and impacted teeth are lower-bound estimates.

The normal prevalence of hypodontia (0.49%) in the presence of extensive malnutrition (73.6% low BMI) suggests that the development of tooth number may be relatively resistant to nutritional stress, a finding we tentatively term the ‘Yemen Paradox.’ This observation aligns with the theory of genetic canalization, which posits that certain developmental processes are buffered against environmental perturbations [22]. However, we acknowledge several important caveats. First, as discussed above, our hypodontia prevalence is likely underestimated due to the lack of radiographic confirmation. Second, this is a preliminary observation requiring confirmation through genetic and radiographic studies. Third, alternative explanations include the possibility that the etiology of hypodontia is primarily driven by genetic factors that are less influenced by the nutritional stressors prevalent in this population. We therefore present the ‘Yemen Paradox’ as a testable hypothesis rather than a confirmed phenomenon.”

The strong association between low BMI and ankyloglossia (tongue tie) warrants careful interpretation. While we initially framed low BMI as a potential risk factor for dental anomalies, the direction of causality could be reversed: ankyloglossia is known to impair feeding in infants [29], potentially leading to poor weight gain and subsequent low BMI. This reverse causality is a plausible alternative explanation for our findings. Additionally, unmeasured confounders such as socioeconomic status, maternal nutritional status during pregnancy, consanguinity (prevalence 30–50% in Yemen), and specific micronutrient deficiencies (vitamin D, A, iron, zinc, folate, and calcium) may account for the observed associations.

While BMI was used as a marker of nutritional status, we could not account for other important confounders such as socioeconomic status, maternal nutritional status during pregnancy, consanguinity (prevalence 30–50% in Yemen), and specific micronutrient deficiencies (vitamin D, A, iron, zinc, folate, and calcium), all of which may independently influence both nutritional status and dental development. The cross-sectional design also precludes determination of the direction of causality.

The observed associations between younger age, low BMI, and specific dental anomalies reflect complex epidemiological patterns in this setting, but causal inferences remain limited by the cross-sectional design.

### Strengths and limitations

A primary limitation of this study is the absence of panoramic radiographs (OPGs) due to field logistics in a conflict-affected environment. Consequently, prevalence rates for hypodontia (0.49%), supernumerary teeth (1.1%), and impacted teeth (3.2%) represent lower-bound clinical estimates, as unerupted anomalies or true agenesis could not be radiographically confirmed. Additionally, while low BMI served as an indicator of nutritional stress, micronutrient deficiencies and genetic consanguinity were not directly measured, and the cross-sectional design precludes causal conclusions. Regarding the statistical analysis, although the intra-cluster correlation coefficient was low and the use of Generalized Estimating Equations (GEE) with cluster-robust standard errors accounted for the sampling design, we acknowledge that some bivariate analyses were not cluster-adjusted. However, the negligible clustering effect minimizes the potential for bias in these exploratory comparisons.

## Conclusions

The prevalence of dental anomalies in Aden, Yemen, was relatively high compared to most global studies. Those who were males, aged less than 10 years and with low BMI had a higher prevalence of DAs. The most frequent anomalies among schoolchildren were tongue tie and macrodontia. Tongue tie was significantly associated with male sex and children aged below 10 years. Furthermore, impacted teeth and supernumerary teeth were associated with children aged below 10 years, while hypodontia, macrodontia, and talon cusps were significantly associated with children in the age group 10–11 years. Low BMI was strongly correlated with supernumerary teeth, talon cusps, and tongue tie. High rates of tongue-tie and impacted teeth, especially among malnourished and younger patients, indicate severe developmental problems due to nutritional stress caused by prolonged warfare. Future investigations with radiographic diagnosis and genetic evaluation are needed.

The ‘Yemen Paradox’—where tooth number anomalies are relatively low in the face of severe malnutrition—is proposed as a hypothesis for further investigation. This is a preliminary observation based on clinical examination alone, and further studies with radiographic diagnosis and genetic evaluation are needed to confirm and refine this observation.”

## Data Availability

All data was presented in the manuscript, no further data available and it will be available at any time.
